# A systematic review of the literature: Gender-based violence in the construction and natural resources industry

**DOI:** 10.3934/publichealth.2024033

**Published:** 2024-05-08

**Authors:** Joyce Lo, Sharan Jaswal, Matthew Yeung, Vijay Kumar Chattu, Ali Bani-Fatemi, Aaron Howe, Amin Yazdani, Basem Gohar, Douglas P. Gross, Behdin Nowrouzi-Kia

**Affiliations:** 1 Department of Occupational Science and Occupational Therapy, University of Toronto, Toronto, ON M5G 1V7, Canada; 2 Center for Global Health Research, Saveetha Medical College and Hospital, Saveetha Institute of Medical and Technical Sciences (SIMATS), Saveetha University, Chennai 600077, India; 3 Department of Community Medicine, Faculty of Medicine, Datta Meghe Institute of Medical Sciences, Wardha 442107, India; 4 Canadian Institute for Safety, Wellness & Performance, School of Business, Conestoga College Institute of Technology and Advanced Learning, Kitchener, ON N2G 4M4, Canada; 5 Department of Population Medicine, University of Guelph, Guelph, ON N1G 2W1, Canada; 6 Centre for Research in Occupational Safety & Health, Laurentian University, Sudbury, ON P3E 2C6, Canada; 7 Department of Physical Therapy, University of Alberta, Edmonton, AB, T6G 2G4, Canada

**Keywords:** construction, gender-based violence, natural resources, systematic review, workplace

## Abstract

Gender-based violence (GBV) poses a significant concern in the construction and natural resources industries, where women, due to lower social status and integration, are at heightened risk. This systematic review aimed to identify the prevalence and experience of GBV in the construction and natural resources industries. A systematic search across databases including PubMed, OVID, Scopus, Web of Science, and CINAHL was conducted. The *Risk of Bias Instrument for Cross-sectional Surveys of Attitudes and Practices* by McMaster University and the *Critical Appraisal of Qualitative Studies* by the Center for Evidence Based Medicine at the University of Oxford were used to assess the studies included in the review. Six articles were included after full-text analysis. GBV was reported in the construction, mining, urban forestry, and arboriculture sectors. Workplace GBV was measured differently across the studies, and all studies examined more than one form of GBV. The main forms of GBV discussed in these studies were discrimination, sexual harassment, and sexism. The studies provided some insight for demographic factors that may or may not be associated with GBV, such as age, region of work, and number of years working in the industry. The review also suggests that workplace GBV has a negative impact on mental health and well-being outcomes, such as higher levels of stress and lower job satisfaction. The current research has not established the effectiveness of interventions, tools, or policies in these workplaces. Thus, additional research should include intervention studies that aim to minimize or prevent GBV in male-dominated workplaces. The current study can bring awareness and acknowledgement towards GBV in the workplace and highlight the importance of addressing it as this review outlines the negative consequences of GBV on mental health and well-being in these male-dominated industries.

## 1. Introduction

Gender-based violence (GBV) can be defined as harmful acts committed against a person because of their gender [1]. It is recognized as a serious violation of human rights and a life-threatening health issue [2]. GBV can include “any word, action, or attempt to degrade, control, humiliate, intimidate, coerce, deprive, threaten, or harm another person” [1]. GBV can occur in the workplace and can take on various forms such as physical abuse, sexual harassment, verbal abuse, bullying, discrimination, coercion, psychological abuse, and abusive working conditions [1],[3]. Although anyone can be a victim of GBV, women and girls are particularly at risk [2]. Across the world, 35% of women are victims of direct violence at work [3]. Data from a 2020 survey found that among Canadian workers, 25% of women and 17% of men reported that they personally experienced inappropriate sexualized behaviors at work during the previous year [4]. Moreover, one in ten women personally experienced workplace discrimination based on their gender, gender identity, or sexual orientation, compared to less than one in twenty men [4]. The same survey also reported that women working in occupations historically dominated by men were more often targeted with inappropriate sexualized behaviors. For example, 47% of women working in equipment operation, trades, transportation, and related occupations have experienced these behaviors at work in the past year [4]. While GBV is a pervasive issue across various sectors, this systematic review narrows its focus to the construction and natural resources industries, shedding light on the unique challenges faced by women in these traditionally male-dominated workplaces.

Male-dominated industries are those in which women only make up to 30% of the workforce [5]. The construction (e.g., skilled trades such as electricians and plumbers) and natural resources (e.g., mining and forestry) sectors are both examples of traditionally male-dominated workplaces [6]. The underrepresentation of women in these sectors needs to be addressed as women's minority status in these industries places them at greater risk of experiencing GBV. This can be explained by women's lower social status and social integration in these industries [7]. For example, organizations that have gendered expectations or tasks also have higher rates of gender-based violence as women are often perceived as less qualified than men for male-typed roles [8]–[10]. Moreover, in traditionally masculine occupations where physical strength is required, women experience greater sexual harassment than in workplaces that do not require physical strength [11]–[13].

Gender-based violence can have significant negative impacts on an individual's physical (e.g., infections and injuries) and mental health (e.g., depression, anxiety, and suicide attempts) [3]. In addition, there are direct and indirect social and economic costs, such as decreased productivity, increased absenteeism, and increased employee turnover [14]. In addition to tangible economic costs, GBV may also perpetuate inequalities and negative stereotypes regarding women's ability to fully participate in the workplace [15]. Other areas of cost resulting from GBV include justice (e.g., legal support), social services (e.g., counselling), and personal costs (e.g., lost earnings from time off work) [16]. According to the International Labour Organization, workplace stress and violence losses are estimated to be 1% to 3.5% of the Gross Domestic Product [17]. Given these rates of GBV in the workplace and the associated negative consequences, understanding how GBV impacts women employees has important implications (e.g., improving employee health and well-being, and addressing skill shortages). To the best of our knowledge, no study has reviewed the literature on the prevalence and impact of workplace GBV in the construction and natural resources industries. Thus, this systematic review seeks to fill a critical gap in the existing literature by examining the prevalence and impact of GBV in the construction and natural resources industries.

## 2. Materials and methods

This systematic review is part of a larger systematic review (PROSPERO CRD42023399684). The current review focuses on identifying the prevalence and experience of GBV, specifically in the construction and natural resources industry.

### 2.1. Search strategy and study selection

A comprehensive search was conducted using a systematic approach, following the Preferred Reporting Items for Systematic Review and Meta-Analysis (PRISMA) guidelines [18]. The search strategy was developed using the following databases: PubMed, OVID, Scopus, Web of Science, and CINAHL. The study's research questions were used to guide the search terms related to “gender”, “GBV”, “workplace”, “skilled trades”, and “industry” (please see Appendix A for the full search strategy). The three team members completed multiple independent searches. The search criteria were formulated utilizing the Population/Intervention/Comparisons/Outcomes (PICO) framework. Inclusion criteria were (1) peer-reviewed original research articles, such as but not limited to experimental and observational studies; (2) published in English; (3) published over the past 10 years (January 2013 to February 2023); and (4) contained findings on workplace GBV for adults (aged 18–65) working in the construction or natural resources (e.g., mining and forestry) sector. Various forms of GBV were considered, including but not limited to discrimination, harassment, and physical, psychological, emotional, societal, economic, and sexual violence [1]. All sex and gender study samples were included (including the LGBTQ+ community). The exclusion criteria were (1) publications such as book chapters, grey literature, reviews, case reports, commentaries, editorials, and conferences; (2) publications in other languages; and (3) GBV that is not work-related/workplace-related or primarily occurring in the work environment (i.e., domestic violence, spousal violence, violence by live-in partners, and self-harm/self-injury).

The search was conducted in accordance with the Peer Review of Electronic Search Strategies (PRESS) statement [19]. Articles retrieved from the search were uploaded and stored in Covidence to be compiled, organized, and evaluated. Four team members were involved in screening the articles, which was conducted in stages. The title and abstract screening process was completed independently by three reviewers and one senior member in consultation with the research team. After this stage, the studies underwent a full-text review to ensure that the inclusion criteria were met. Any disagreements regarding a study's inclusion were discussed, and a consensus was reached among the team.

### 2.2. Data analysis and quality assessment

Quality assessment was conducted by four reviewers (JL, SJ, MY, and VKC). Critical appraisal tools were used, based on the typology of the study. For cross-sectional studies, the *Risk of Bias Instrument for Cross-sectional Surveys of Attitudes and Practices* by McMaster University [20] was used. The same instrument was used for the mixed-methods study in addition to the *Critical Appraisal of Qualitative Studies* developed by the Center for Evidence Based Medicine at the University of Oxford [21]. No studies were excluded based on the quality assessment.

A narrative synthesis was conducted to summarize the findings from the included studies. A meta-analysis was not performed due to heterogeneity in study designs and outcome measures.

## 3. Results

**Figure 1.**
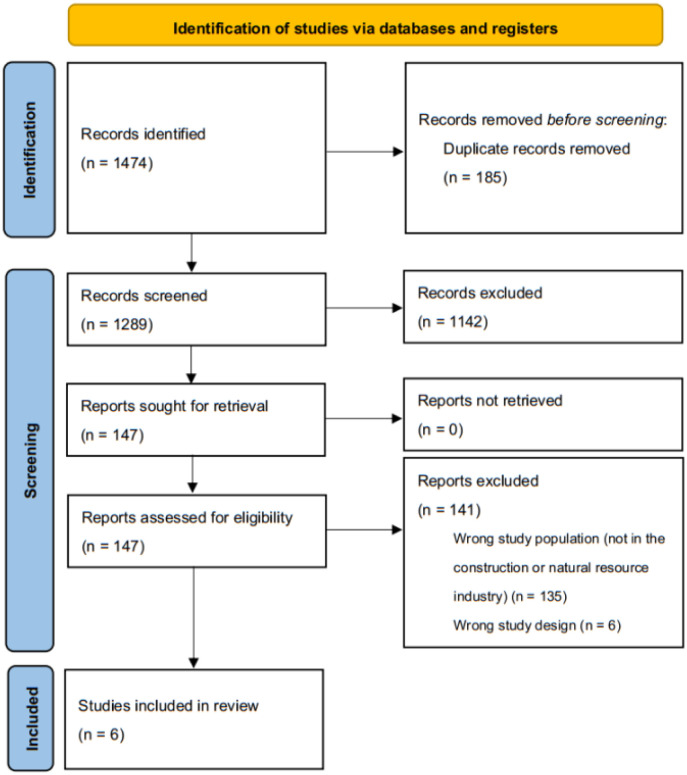
The Preferred Reporting Items for Systematic Review and Meta-Analysis (PRISMA) diagram.

The initial search for the larger systematic review yielded 1474 studies, of which 186 duplicates were removed. From the results of the larger systematic review, the current review excluded 141 full texts, only including the studies that focused on GBV in the construction and natural resources sectors. Six articles were included in this review after a full-text analysis. Figure 1 outlines the article selection process.

### 3.1. Descriptive overview

Table 1 provides a summary of the studies included in this review. The studies included in the review contained information on workplace GBV experiences in the construction and natural resources sectors. Half of the studies were conducted in Australia (*n* = 3), followed by Canada and the United States (*n* = 1), South Africa (*n* = 1), and South Korea (*n* = 1). GBV was reported in the construction, mining, and urban forestry and arboriculture sectors. Half of the studies focused on women working in these sectors. The sample sizes ranged from 85 to 626 participants. All studies were cross-sectional, with five studies using quantitative research methods and one study using a mixed-methods approach. There were no common instruments used to measure GBV in the workplace. The main forms of GBV discussed in these studies were discrimination, sexual harassment, and sexism. One study reported perpetrators of gender discrimination, including line managers (8%) and colleagues (10%) [22]. Another study revealed that women working in forestry experienced discrimination from both women and men [23].

**Table 1.** Summary of the included full-texts as per the eligibility criteria (*n* = 6).

**First author**	**Year**	**Country**	**Sample size; target population**	**Study design**
Bowen et al. [22]	2013	South Africa	*n* = 626; Construction professionals	Cross-sectional
Park et al. [24]	2022	Korea	*n* = 85; Health managers in construction	Cross-sectional
Sunindijo et al. [25]	2017	Australia	*n* = 277; Professionals in the construction industry	Cross-sectional
Rubin et al. [26]	2017	Australia	*n* = 263; Women miners	Cross-sectional
Bardekjian et al. [23]	2019	Canada and United States	*n* = 515; Women in urban forestry and arboriculture	Cross-sectional, Mixed methods
Rubin et al. [27]	2019	Australia	*n* = 190; Women in trades	Cross-sectional

### 3.2. Quality assessment of included studies

The critical appraisal domain scores for the included cross-sectional studies are presented in Table 2. Common domains with high or unclear risk of bias include inadequate response rates, lack of reliable or valid survey instruments, and unreported rates of missing data. One mixed-methods study by Bardekjian et al. (2019) scored a low risk of bias for nine out of 13 domains [23]. The remaining domains were unclear.

**Table 2.** Quality assessment of cross-sectional studies using the Risk of Bias Instrument for Cross-sectional Surveys of Attitudes and Practices.

**First author**	**Is the response rate adequate?**	**Is the source population representative of the population of interest?**	**Is the survey clinically sensible?**	**Is there any evidence for the reliability and validity of the survey instrument?**	**Is there little missing data?**
Bowen et al. 2013 [22]	High	Low	Low	Unclear	Unclear
Park et al. 2022 [24]	High	Low	Unclear	High	Unclear
Rubin et al. 2017 [26]	High	High	Low	High	Low
Rubin et al. 2019 [27]	Low	Low	Low	Low	Low
Sunindijo et al. 2017 [25]	Low	Low	Unclear	Unclear	Unclear

### 3.3. Demographic factors and GBV

Demographic differences have also been reported in this regard. Three studies that included a mixed sample of men and women found that women experienced GBV more often than men did. For example, Park et al. (2022) indicated that female health managers in construction reported significantly higher rates of sexual harassment than their male counterparts (59.7% versus 15.4%) [24]. Similarly, Sunindijo et al. (2017) indicated that women in construction experience ethics-related issues (i.e., sexual harassment, differential treatment, and bullying) more often than men [25]. One study reported that older women working in mining and women working in Australian mining sites (compared to African, South American, and Southeast Asian sites) reported lower levels of organizational and interpersonal sexism [26]. Their findings also suggest that the number of years of work in the industry is not significantly associated with organizational and interpersonal sexism. Another study found that female construction health managers with more than two years of career experience had higher response rates for sexual harassment, verbal abuse, and physical violence than those with less than two years of career experience [24]. Bardekjian et al. (2019) found that participants with a moderate level of work experience in the urban forestry and arboriculture sector (7–10 years) were less likely to agree that women and men are treated equally in their industry compared to those with extensive (11+ years) and limited experience (6 years or less) [23].

### 3.4. Prevalence and types of GBV

Workplace GBV was measured differently across the studies, and all studies examined more than one form of GBV. The main forms of workplace GBV include discrimination, harassment, and sexism. Some forms of harassment, specifically sexual harassment, were measured across all studies. The two studies by Rubin and colleagues measured gender-based workplace issues labeled as interpersonal sexism and organizational sexism [26],[27]. Other studies looked at more specific forms of sexism, including the gender pay gap and feeling underpaid due to gender [22],[23], feelings of job insecurity due to gender [22], and inappropriate work orders [24].

Prevalence rates were also reported. Bowen et al. (2013) found that among construction professionals, 8% of respondents indicated they had experienced harassment from colleagues because of their gender [22]. Respondents also reported discrimination from their line managers (8%) and colleagues (10%) because of their gender. Ten percent of respondents felt underpaid because of their gender and 13% reported feelings of job insecurity because of their gender. Another study revealed that inappropriate work orders were the most reported form of workplace violence for female health managers in construction (84.7%), followed by sexual harassment (59.7%), verbal abuse (48.6%), and physical violence (5.6%) [24]. In a study focusing on women in forestry and arboriculture, 60% of participants reported that women and men were not treated equally in their industry and 84% agreed that women in their industry face gender-based challenges [23]. Additionally, 74% reported that they have experienced or witnessed sexist behavior or sexual harassment in their industry.

### 3.5. Impacts of GBV

Four studies measured mental health and well-being as the outcomes. For example, Bowen et al. (2013) found that respondents who experienced discrimination based on gender reported higher levels of stress [22]. The authors also reported that participants felt underpaid and felt job insecurity because of their gender. Rubin et al. (2017) found that in women miners, both organizational and interpersonal sexism were negatively related to sense of belonging and job satisfaction and positively related to mental health problems [26]. The same researchers conducted another study in 2019 with women in mining, construction, and forestry, confirming most of their previous findings and identifying a sense of belonging as mediating the associations between organizational sexism and mental health and job satisfaction [27]. However, contrary to their previous study, interpersonal sexism did not predict job satisfaction.

### 3.6. Interventions for GBV

There were no intervention studies included. However, one study discussed overcoming barriers [23]. Women in forestry and arboriculture indicated strategies to overcome workplace barriers: sponsorship/mentoring, confidence, communication, work-life balance, and career planning.

## 4. Discussion

The objective of this systematic review was to identify the prevalence and experience of GBV in male-dominated industries, more specifically, the construction and natural resources sector. In this systematic review, we included six articles which contained information on GBV in construction and natural resources workplaces. The research suggests that GBV is prevalent in these workplaces. The studies provided some insight for demographic factors that may or may not be associated with GBV, such as age, region of work, and the number of years working in the industry. The review also identified common forms of workplace GBV in the construction and natural resources industry, which include discrimination, harassment, and sexism. Furthermore, the review suggests that workplace GBV has a negative impact on mental health and well-being outcomes, such as higher levels of stress and lower job satisfaction.

Building upon the current findings, it is noteworthy to consider insights from a recent systematic review by Riddle and Heaton (2023), which looked at antecedents to the sexual harassment of women in male-dominated industries, including law enforcement, firefighting, truck driving, and construction [28]. Their review suggests that organizational culture and gender composition are primary antecedents to sexual harassment in these occupations, which may explain some of the findings in the present review. Studies in the current review mostly discuss findings related to workplace relationships. For example, for women in forestry and arboriculture, Bardekjian et al. (2019) identified discrimination and workplace relationship barriers, including microaggressions, not being taken seriously [23]. When looking at prevalence rates in other male-dominated industries, reported rates of GBV seem to be higher in women compared to men. A literature review on workers in the maritime industry found that workplace bullying and harassment prevalence rates ranged from 8% to 25% of all seafarers and over 50% of women seafarers [29]. Similar findings were reported in a study looking at GBV within orthopedic surgery in Canada, as women were 16.2 times more likely than men to report experiencing an instance of gender-based harassment and 2.2 times more likely to report experiencing an instance of sexual harassment [30]. Given these prevalence rates, this may be a contributing factor to the prevailing gender disparity in these fields.

The review found negative consequences associated with experiencing workplace GBV in the construction and natural resources industries, including higher stress and lower job satisfaction. The negative effects of sexual harassment have been documented across many industries and occupations [31]. For example, a systematic review that looked at workplace violence among healthcare workers found that victims of workplace violence have lower levels of job satisfaction compared to non-victims [32]. There is also evidence for the relationship between experiencing GBV and reporting negative psychological consequences (e.g., worse mental health) among healthcare workers. Studies report similar findings in samples with university employees [33],[34], police officers [35], and military personnel [36].

There was variety in the origin of the study and sample setting. Studies originated from multiple countries, suggesting that GBV in these workplaces is a global problem. However, none of the included studies evaluated interventions or policies to address GBV in the workplace. A previous systematic review by Diez-Canseco et al. (2022) found limited literature regarding the effectiveness of policies and training to prevent sexual harassment in the workplace [37]. Policies and interventions to minimize or prevent GBV in the workplace have not been prioritized in the literature. However, evidence indicates that training and policies can improve awareness, knowledge, and resources to address workplace sexual harassment. The current study can bring awareness and acknowledgement toward GBV in the workplace and highlight the importance of addressing it. This review outlines the negative consequences of GBV on mental health and well-being in the construction and natural resources sectors, reinforcing the need to address this issue. The results may encourage employers to improve their work environment by implementing interventions, creating policies surrounding GBV (e.g., how to report GBV), and providing education and training on workplace GBV.

More advanced research designs, such as correlational and intervention studies, are needed. Further research should explore how the implementation of different policies impacts GBV rates over time to inform continued improvement in the workplace setting. For example, to reduce workplace sexism among female workers in male-dominated industries, creating an intervention or program that improves the sense of belonging in the workplace could be investigated, as suggested by the findings of Rubin et al. (2019) [27]. Due to the lack of literature on GBV interventions in the workplace, findings from other violence intervention studies may help inform future research methods. For example, the literature suggests that educational interventions may be effective. Findings from an integrative review on workplace violence against home healthcare workers suggest that safety and health training is effective in reducing workplace violence incidents and increasing confidence and knowledge about workplace violence [38]. Similarly, a systematic review on workplace interventions for intimate partner violence mainly looked at educational interventions and found that benefits may include increased knowledge of intimate partner violence and policies, willingness to intervene, and provision of information and resources to intimate partner violence victims [39].

### 4.1. Limitations

One limitation identified by this review is that it cannot avoid or correct the biases that already exist in the studies that were included in this systematic review. For example, a few studies that were included indicated self-report and response bias as limitations of their study. Another limitation was that only articles published in English between January 2013 to February 2023 were included. This period was selected to encompass the most recent and pertinent contributions to the understanding of workplace GBV in the construction and natural resources sectors. However, we may have excluded studies with relevant information outside these criteria. It should also be noted that we included studies with workers in both on-site and off-site (e.g., administration) positions. The differences between these workers were not explored in this review as most of the studies included did not compare these groups. In this review, our definition of GBV is broad because part of our objective was to identify experiences of GBV, which has various forms. Due to the lack of consistency in terms of describing and measuring GBV, there is significant difficulty in cross-comparing studies, limiting the generalizability of the findings. Additionally, in using a broad definition of GBV, this may be seen as a limitation in our search terms. Lastly, we excluded studies of violence that were not work-related/workplace-related or primarily occurring in the work environment (e.g., domestic violence). Although we acknowledge that these forms of GBV can occur in and impact the workplace, we decided to exclude these studies to focus on GBV that stems from work-related/workplace-related factors.

## 5. Conclusions

This systematic review examines the literature on the prevalence and impact of workplace GBV in the construction and natural resources sectors. Although women's participation in these sectors is increasing, this review underscores the persistent prevalence of workplace GBV in the construction and natural resources sectors. This review highlights the negative impact of GBV on mental health and well-being in these groups, as well as the lack of intervention studies in the literature. Thus, future research should include the implementation of interventions that aim to minimize or prevent GBV in male-dominated workplaces. The imperative to create safer, more equitable work environments remains a shared responsibility for employers, policymakers, and industry stakeholders alike.

## Use of AI tools declaration

The authors declare they have not used Artificial Intelligence (AI) tools in the creation of this article.


