# Supplementary material for: A systematic review of the literature: Gender-based violence in the construction and natural resources industry
Source: AIMS Public Health. 2024 May 8;11(2):654–66. doi: 10.3934/publichealth.2024033 (PMC11252573; doi:10.3934/publichealth.2024033)
Supplement: Supplementary file 1 [file publichealth-11-02-033-s001.pdf]

Public Health

AIMS Public Health, 11(2): 654–666.
DOI: 10.3934/publichealth.2024033
Received: 08 February 2024
Revised: 09 April 2024
Accepted: 12 April 2024
Published: 08 May 2024

https://www.aimspress.com/journal/aimsph

Review

A systematic review of the literature: Gender-based violence in the construction and natural resources industry

Joyce Lo[1], Sharan Jaswal[1], Matthew Yeung[1], Vijay Kumar Chattu[1,2,3], Ali Bani-Fatemi[1], Aaron Howe[1], Amin Yazdani[4], Basem Gohar[5,6], Douglas P. Gross[7], and Behdin Nowrouzi-Kia[1,6,*]

[1] Department of Occupational Science and Occupational Therapy, University of Toronto, Toronto, ON M5G 1V7, Canada
[2] Center for Global Health Research, Saveetha Medical College and Hospital, Saveetha Institute of Medical and Technical Sciences (SIMATS), Saveetha University, Chennai 600077, India
[3] Department of Community Medicine, Faculty of Medicine, Datta Meghe Institute of Medical Sciences, Wardha 442107, India
[4] Canadian Institute for Safety, Wellness & Performance, School of Business, Conestoga College Institute of Technology and Advanced Learning, Kitchener, ON N2G 4M4, Canada
[5] Department of Population Medicine, University of Guelph, Guelph, ON N1G 2W1, Canada
[6] Centre for Research in Occupational Safety & Health, Laurentian University, Sudbury, ON P3E 2C6, Canada
[7] Department of Physical Therapy, University of Alberta, Edmonton, AB, T6G 2G4, Canada

* **Correspondence**: Email: behdin.nowrouzi.kia@utoronto.ca; Tel: +14169463249.

Supplementary

APPENDIX A. Databases and search results

CINAHL – 305 articles

1. (Gender-based violence or GBV or Harassing or Harassment or Cyberbullying or Cyberhate or Cyberharassment or Gender discrimination or violence victim or exploitation or bullying or intimidation)
2. [(Virtual or web or Online or Remote or Digital or Internet or telecommuting or teleworking)] AND [(work or employment or labour or workplace)]
3. (university or academia or college or healthcare or Skilled trades or industry)
4. S1 AND S2
5. S3 AND S4

OVID – 361 articles

1. (Gender-based violence or GBV or Harassing or Harassment or Cyberbullying or Cyberhate or Cyberharassment or Gender discrimination or violence victim or exploitation or bullying or intimidation).tw,kf.
2. [(Virtual or web or Online or Remote or Digital or Internet or telecommuting or teleworking) adj2 (work or employment or labour or workplace)].tw,kf.
3. (university or academia or college or healthcare or Skilled trades or industry).tw,kf.
4. 1 and 2
5. 3 and 4

PubMed – 122 articles

(“university”[Title/Abstract] OR “academia”[Title/Abstract] OR “college”[Title/Abstract] OR “healthcare”[Title/Abstract] OR “skilled trades”[Title/Abstract] OR “industry”[Title/Abstract]) AND (“gender based violence”[Title/Abstract] OR “GBV”[Title/Abstract] OR “Harassing”[Title/Abstract] OR “Harassment”[Title/Abstract] OR “Cyberbullying”[Title/Abstract] OR “Cyberhate”[Title/Abstract] OR “Cyberharassment”[Title/Abstract] OR “gender discrimination”[Title/Abstract] OR “violence victim”[Title/Abstract] OR “exploitation”[Title/Abstract] OR “bullying”[Title/Abstract] OR “intimidation”[Title/Abstract]) AND ((“Virtual”[Title/Abstract] OR “web”[Title/Abstract] OR “Online”[Title/Abstract] OR “Remote”[Title/Abstract] OR “Digital”[Title/Abstract] OR “Internet”[Title/Abstract] OR “telecommuting”[Title/Abstract] OR “teleworking”[Title/Abstract]) AND (“work”[Title/Abstract] OR “employment”[Title/Abstract] OR “labour”[Title/Abstract] OR “workplace”[Title/Abstract]))

Scopus - 57 articles

[(TITLE-ABS-KEY (“gender-based violence” OR gbv OR harassing OR harassment OR cyberbullying OR cyberhate OR cyberharassment OR “gender discrimination” OR “violence victim” OR exploitation OR bullying OR intimidation)] AND [TITLE-ABS-KEY (virtual OR web OR online OR remote OR digital OR internet OR telecommuting OR teleworking W/2 work OR employment OR labour OR workplace)] AND [TITLE-ABS-KEY (university OR academia OR college OR healthcare OR “Skilled trades” OR industry)]

AND [LIMIT-TO (PUBYEAR, 2023) OR LIMIT-TO (PUBYEAR, 2022) OR LIMIT-TO (PUBYEAR, 2021) OR LIMIT-TO (PUBYEAR, 2020) OR LIMIT-TO (PUBYEAR, 2019) OR LIMIT-TO (PUBYEAR, 2018) OR LIMIT-TO (PUBYEAR, 2017) OR LIMIT-TO (PUBYEAR, 2016) OR LIMIT-TO (PUBYEAR, 2015) OR LIMIT-TO (PUBYEAR, 2014) OR LIMIT-TO (PUBYEAR, 2013)]

Web of science – 629 articles

1. TS=(Gender-based violence or GBV or Harassing or Harassment or Cyberbullying or Cyberhate or Cyberharassment or Gender discrimination or violence victim or exploitation or bullying or intimidation)
2. TS=(Virtual or web or Online or Remote or Digital or Internet or telecommuting or teleworking) AND (work or employment or labour or workplace)
3. TS=(university or academia or college or healthcare or Skilled trades or industry)
4. #1 AND #2
5. #3 AND #4

AIMS Press

© 2024 the Author(s), licensee AIMS Press. This is an open access article distributed under the terms of the Creative Commons Attribution License (https://creativecommons.org/licenses/by/4.0).
